# Vaccination Hesitancy and Its Impact on Immunization Coverage in Pediatrics: A Systematic Review

**DOI:** 10.7759/cureus.76472

**Published:** 2024-12-27

**Authors:** Sahar Ali Osman Mohamed Elawad, Azza Abdelbagi Yagoub Mohammed, Samia Ahmed Ali Karar, Aala Abdelrahman Hassan Farah, Ahmed Mohamed Elamin Mubarak Osman

**Affiliations:** 1 Pediatrics, Armed Forces Hospital, Jazan, SAU; 2 Neonatal Intensive Care, Maternity and Children Hospital, Hail, SAU; 3 Pediatrics, Royal Glamorgan Hospital, Cardiff, GBR; 4 Pediatric Medicine, Leeds Teaching Hospitals National Health Service (NHS) Trust, Leeds Children’s Hospital, Leeds, GBR; 5 General Medicine, Jouf University Medical Services Center, Jouf, SAU

**Keywords:** immunization, pediatric, systematic review, vaccination coverage, vaccination hesitancy

## Abstract

One significant global health issue that is present in more than 190 nations globally is routine vaccination reluctance. This study aimed to synthesize the current evidence on vaccination hesitancy and its impact on immunization coverage in pediatrics. We searched for relevant studies across four databases (Scopus, Web of Science, PubMed/EMBASE, and Cumulated Index in Nursing and Allied Health Literature). Prespecified inclusion and exclusion criteria were used to extract relevant studies while excluding irrelevant ones. We found 4,085 studies on four different databases in which 23 satisfied the inclusion and exclusion criteria. These 23 relevant studies involving 29,131 parents, guardians, and caregivers from over 30 countries met the inclusion criteria and quality assessment. Studies were assessed for risk bias using the Newcastle-Ottawa scale. Vaccination hesitancy is caused by several factors, such as cultural customs, economic reforms, perceived rumors, myths, misconceptions, physicians and other healthcare professionals, and perceived risks and problems of vaccines. These results highlight the importance of addressing demand-side factors related to socioeconomic determinants and supply-side issues such as improving health literacy, combating misinformation, ensuring clarity in communication, and promoting a consistent, evidence-based message. More observations and research should be conducted regularly to develop strategies for encouraging youngsters to receive immunizations in large quantities.

## Introduction and background

Vaccination is regarded as one of the most significant advancements in public health [1]. Vaccination programs are credited with eradicating smallpox and poliomyelitis worldwide, as well as decreasing the morbidity and mortality of other infectious diseases. For instance, polio was eradicated in India in 2014 despite challenges such as high population density and remote, hard-to-reach areas. Similarly, the African region was certified polio-free in 2020, overcoming significant barriers in conflict-affected and resource-limited settings. High levels of vaccination program uptake are necessary for the incidence and prevalence of vaccine-preventable diseases (VPDs) to be successfully decreased [2]. High vaccination rates not only provide direct protection for those who receive vaccinations but also lower the risk of infection for those who are still vulnerable in the community by reducing the spread of VPD, a phenomenon known as herd immunity [3].

The fact that the majority of affluent nations have high childhood immunization rates, with markedly reduced incidence of VPDs compared to where immunization rates are low, suggests that vaccination is still a commonly used public health intervention. These national figures, however, might conceal populations with low vaccination rates [4]. Undervaccinated or unvaccinated populations have been primarily blamed for recent VPD epidemics, which have included measles, poliomyelitis, and pertussis in various affluent nations. Furthermore, numerous research findings have demonstrated that even those who have had vaccinations may harbor significant reservations and worries about them [5-7].

Parental vaccine hesitation regarding children's vaccinations is one specific issue. Children and the communities around them suffer greatly when parents are reluctant to vaccinate, as VPDs remain a significant source of morbidity and mortality. For instance, the World Health Organization (WHO) reported that, in 2019, measles caused over 207,500 deaths globally, with outbreaks being disproportionately severe in undervaccinated regions. Conversely, highly vaccinated communities have seen dramatic reductions in disease incidence and related deaths, underscoring the importance of widespread immunization efforts. About one in four parents in the United States had grave reservations about vaccinating their children, according to a 2019 nationwide study. While overall childhood immunization rates stayed high between 2012 and 2017, the number of children who were not vaccinated at the age of 24 months continued to rise, according to a 2018 report from the Centers for Disease Control and Prevention (CDC) [3,8]. More than one-third of American children aged 19-35 months were not adhering to the advised early childhood immunization schedule, according to a March 2020 analysis of the most recent CDC National Immunization Survey data [9].

Understanding the impact of vaccination hesitancy on pediatric immunization coverage requires a comprehensive and systematic analysis of the available evidence. The reasons for hesitancy vary widely across different settings and populations, necessitating a nuanced approach to explore the underlying causes and their consequences. Furthermore, this issue is further compounded by the rapid dissemination of misinformation through social media platforms, which has been shown to amplify parental concerns and distrust toward vaccines. Identifying and addressing these challenges is essential to design effective interventions that can mitigate hesitancy and improve vaccine uptake [10].

This systematic review aims to synthesize the current evidence on vaccination hesitancy and its impact on immunization coverage in pediatrics. By critically examining studies from diverse geographical and cultural contexts, this review seeks to provide a comprehensive understanding of the extent of the problem, its driving factors, and its implications for public health policies. Ultimately, the findings of this review will contribute to developing targeted strategies aimed at enhancing vaccination acceptance and ensuring the sustainability of immunization programs for children worldwide.

## Review

Methodology

Study Design

The systematic review adhered to the Preferred Reporting Items for Systematic Reviews and Meta-Analyses [11] technical checklists to enhance the transparency and usability of the approach.

Search Strategy

Without considering the publishing date, we looked through four (Scopus, Web of Science, PubMed/EMBASE, and Cumulated Index in Nursing and Allied Health Literature, CINAHL) databases to find studies that were published in English. Furthermore, we searched these databases for any recent or earlier systematic reviews on the topic. EndNote software (Clarivate, London, UK) was used to merge the results from four datasets and remove duplicates. Gray literature was excluded to ensure the inclusion of only high-quality, peer-reviewed studies, minimizing the risk of bias and inaccuracies. A list of the databases and search methods used is provided in Table 1.

**Table 1:** Search strategies used for different databases CINAHL: Cumulated Index in Nursing and Allied Health Literature

Sr. no.	Database	Search string
1	Scopus	(TITLE-ABS-KEY("vaccination hesitancy" OR "vaccine hesitancy" OR "vaccine refusal" OR "vaccine acceptance" OR "immunization delay") AND ("immunization coverage" OR "vaccination coverage" OR "vaccine uptake" OR "immunization rates") AND ("pediatrics" OR "children" OR "infants" OR "adolescents"))
2	Web of Science	TS=("vaccination hesitancy" OR "vaccine hesitancy" OR "vaccine refusal" OR "vaccine acceptance" OR "immunization delay") AND ("immunization coverage" OR "vaccination coverage" OR "vaccine uptake" OR "immunization rates") AND ("pediatrics" OR "children" OR "infants" OR "adolescents")
3	PubMed/EMBASE	(("vaccination hesitancy"[Title/Abstract] OR "vaccine hesitancy"[Title/Abstract] OR "vaccine refusal"[Title/Abstract] OR "vaccine acceptance"[Title/Abstract] OR "immunization delay"[Title/Abstract]) AND ("immunization coverage"[Title/Abstract] OR "vaccination coverage"[Title/Abstract] OR "vaccine uptake"[Title/Abstract] OR "immunization rates"[Title/Abstract]) AND ("pediatrics"[MeSH Terms] OR "children"[MeSH Terms] OR "infants"[MeSH Terms] OR "adolescents"[MeSH Terms]))
4	CINAHL	("vaccination hesitancy" OR "vaccine hesitancy" OR "vaccine refusal" OR "vaccine acceptance" OR "immunization delay") AND ("immunization coverage" OR "vaccination coverage" OR "vaccine uptake" OR "immunization rates") AND ("pediatrics" OR "children" OR "infants" OR "adolescents")

Eligibility Criteria

The inclusion and exclusion criteria for this systematic review are listed below (Table 2). The purpose of this was to determine how common reluctance was among guardians, parents, and other caregivers.

**Table 2:** Inclusion and exclusion criteria

Question elements	Inclusion criteria	Exclusion criteria
Population	Studies focusing on pediatric populations, including children and adolescents (under 18 years of age)	Studies focusing exclusively on adult populations without any relevance to pediatrics
Intervention	Studies addressing vaccination hesitancy, vaccine acceptance, or refusal in relation to childhood immunization	Studies not directly examining vaccination hesitancy or its impact on immunization coverage
Outcomes	Studies reporting immunization coverage, vaccination uptake rates, or the impact of hesitancy on vaccine-preventable disease rates	Studies that do not report measurable outcomes related to immunization coverage, vaccine uptake, or determinants of hesitancy
Study design	Quantitative studies, including observational (cohort, case-control, cross-sectional) and interventional (randomized controlled trials, quasi-experimental) designs	Case reports, commentaries, editorials, or opinion pieces
Study language	Studies published in English	Non-English language publications

Studies Selection

The retrieved articles were saved in EndNote for further use. First, all duplicated records were extracted from the list, and titles and abstracts were reviewed and summed by two chosen authors (SAME and AAYM). Those articles that could potentially meet these criteria were searched in full-text and then cross-checked based on their eligibility against the inclusion and exclusion criteria. Cases of dissimilar scores were discussed and compared to another reviewer (AESI), who served as a tiebreaker. Data from included studies were gathered using a Microsoft® Excel spreadsheet (Microsoft Corporation, Redmond, WA).

Data Extraction

Papers were independently screened by two reviewers (SAME and AAYM), who then ranked them according to the study design, key findings, first author name, and publication year. They extracted individual data on research features, methodology, and outcome measures using a standardized form. Discussions were held to settle the disagreements about the chosen papers by the third reviewer (AESI), who served as a tiebreaker.

Risk Bias Assessment

The risk bias of the included studies was assessed using the Newcastle-Ottawa scale (NOS). To categorize studies as low, moderate, or high, we considered selection process bias, intervention bias, departure from intervention bias, missing data bias, outcome bias, and results bias. Using the inclusion and exclusion criteria, a preference for selection was determined. Performance bias was assessed by considering allocation concealment and using a control arm. Different rankings were given to data management, full industrial sponsorship, biased reporting, and selective reporting. Reviewers looked at eligibility limitations and reported consistency across several sessions. The research was chosen by a second reviewer who considered any disparities in the ratings of the reviewers.

Results

Search Results

**Figure 1:**
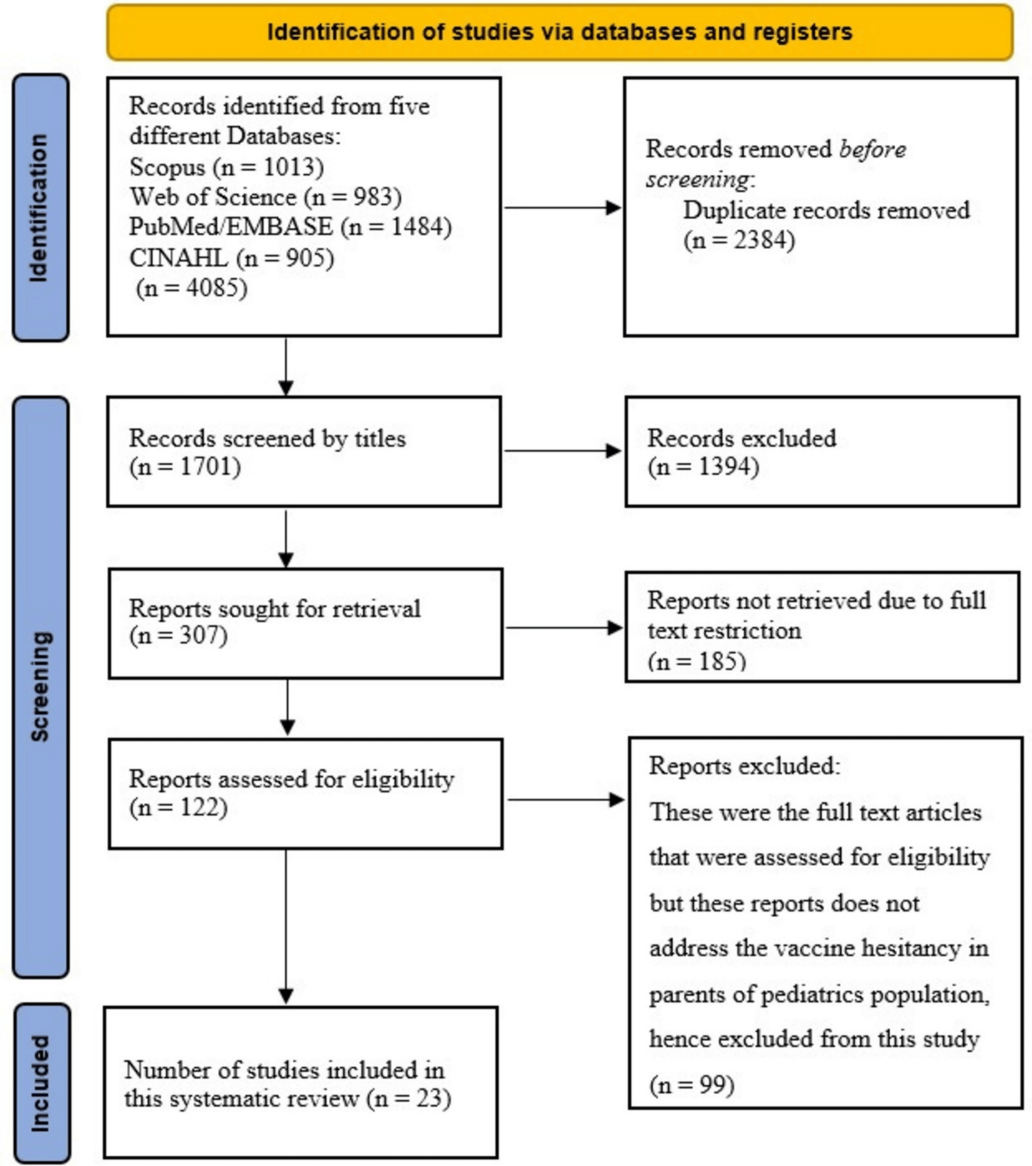
PRISMA flowchart PRISMA: Preferred Reporting Items for Systematic Reviews and Meta-Analyses Source: [12]

A total of 4,085 articles were found through searches in four databases (PubMed, Scopus, Web of Science, and CINAHL). Only 23 of them passed the quality assessment and satisfied the inclusion/exclusion criteria; 29,131 parents, guardians, and other caregivers of children ages 0-6 participated in these 23 reports (Figure 1).

Risk Bias Assessment

NOS was used to assess the risk of bias in the present review. In the 23 retrieved studies, nine had a low bias risk, 13 had a moderate bias risk, and one had a high risk. One of the most significant methodological issues that concerned the authors of the present review related to the choice of controls in some of the investigations. Additionally, all the studies failed to mention blinding controls and patients for exposure, which might have contributed to measurement bias.

For the current systematic review, using the GRADEpro Guideline Development Tool (Evidence Prime Inc., Hamilton, ON), the quality of the evidence was considered moderate across the included studies. Most of these moderate qualities were achieved by sampling a heterogeneity of the included studies and heavily using observational, randomized controlled trials. These designs do not have the component of random exposure inherent to them, which, in turn, increases the exposure to bias (Table 3).

**Table 3:** Risk of bias assessment using the Newcastle-Ottawa scale Rating scale: seven to nine stars = low risk of bias; four to six stars = moderate risk of bias; zero to three stars = high risk of bias Selection: 1 = Is the definition sufficient? 2 = Is the case sufficiently representative? 3 = Select control (hospital or community). 4 = Definitions of controls Comparability: 1 = comparability of cases and controls based on design or analysis Exposure: 1 = determining exposure; 2 = calculation controls and cases are handled using the same methodology; 3 = the rate of nonreaction For every numbered item in the exhibit and selection categories, a study may receive one star (★). No more than two stars (★★) can be awarded for comparability. The hyphen (-) in empty cells signifies that the study has not received a star

Study	Selection				Comparability	Exposure		
	1	2	3	4	1	1	2	3
Brown et al. [13]	★	★	-	-	★★	★	-	★
Voo et al. [14]	★	★	-	-	★	★	★	-
Giambi et al. [15]	★	★	-	-	-	★	★	★
Cherian et al. [16]	★	★	★	-	★★	★	★	★
Campbell et al. [17]	★	★	-	-	★★	★	★	★
Olbrich Neto and Olbrich [18]	★	★	-	-	★	★	★	★
Migriño et al. [19]	★	★	-	-	★★	-	★	★
Bocquier et al. [20]	★	★	-	★	★★	★	★	★
Wang et al. [21]	★	★	★	-	★	-	★	★
Masters et al. [22]	★	★	-	-	★★	★	★	★
Yakum et al. [23]	★	★	-	-	-	-	★	-
Ghosh et al. [24]	★	★	-	-	-	★	★	★
Goruntla et al. [25]	★	★	★	-	★★	★	★	★
Khattak et al. [26]	★	★	★	-	★	★	★	-
Hadjipanayis et al. [27]	★	★	-	-	-	★	★	-
Durmaz et al. [28]	★	★	-	-	★★	★	★	★
Yörük and Güler [29]	★	★	-	-	★	★	★	★
Thapar et al. [30]	★	★	-	★	-	★	★	★
Ustuner Top et al. [31]	★	★	★	-	★★	★	★	★
Dubé et al. [32]	★	★	-	-	★★	★	★	★
Williams et al. [33]	★	★	-	★	★	★	★	★
Sahoo et al. [34]	★	★	-	-	-	★	★	★
Bianco et al. [35]	★	★	★	-	-	★	★	★

Characteristics of Included Studies

This systematic review included 23 studies that investigated vaccination hesitancy and its impact on immunization coverage in pediatrics. All studies employed a cross-sectional design, except one study by Hadjipanayis et al. [27], which utilized a mixed-methods approach. The studies were conducted across various geographical locations, including Brazil [13,18], Malaysia [14], Italy [15,35], India [16,24,25,34], England [17], Philippines [19], France [20], China [21], Ethiopia [22], Cameroon [23], Pakistan [26], 18 European countries [27], Turkey [28,29,31], Canada [32], and the United States [33]. This diverse geographical representation allows for a broader understanding of vaccination hesitancy in different cultural and socioeconomic contexts (Table 4).

**Table 4:** Characteristics and key findings of included studies COVID-19: coronavirus disease 2019; PACV: Parents Attitudes about Childhood Vaccines; SAGE: Strategic Advisory Group of Experts on Immunization; VCI: vaccine confidence index; VH: vaccine hesitancy; WHO: World Health Organization

Study	Publishing year	Country	Study design	Sample size	Study period	Data collection tool	Key findings
Brown et al. [13]	2018	Brazil	Cross-sectional study	952 parents	February and July 2016	VCI questionnaire	Despite the high level of overall vaccination confidence, there was a discernible trend toward lower confidence levels being linked to higher levels of reluctance. Because vaccine hesitancy is dynamic and ever-changing, this calls for ongoing monitoring
Voo et al. [14]	2021	Malaysia	Cross-sectional study	405 parents	February-March 2018	Self-administered questionnaire	Higher educated parents were less vaccine-apprehensive, knew more about vaccinations, and were more inclined to make sure their kids finished the prescribed vaccination schedule, according to the study
Giambi et al. [15]	2018	Italy	Cross-sectional study	3,130 parents	December 2015 to June 2016	Self-structured questionnaire	All parents are concerned about vaccine safety, but reluctant and antivaccine parents are more so. Reluctant parents trust their own pediatricians and view vaccination as a preventative measure, much like provaccine parents do. This suggests that they might benefit from effective communication strategies
Cherian et al. [16]	2022	India	Cross-sectional study	350 caregivers	November 2015 to April 2017	Self-structured questionnaire	It was discovered that vaccine reluctance was quite common. The World Health Organization identified vaccination hesitancy as one of the main obstacles to improved global health in 2019
Campbell et al. [17]	2017	England	Cross-sectional study	1,792 parents	January and April 2015	Self-administered questionnaire	Parents continue to place a high value on health professionals in providing information on vaccinations, and this trust has grown in recent years
Olbrich Neto and Olbrich [18]	2023	Brazil	Cross-sectional study	1,261 parents	January 2018 to December 2019	Self-administered questionnaire	Parents believe that vaccines are safe and effective at preventing disease, and have more advantages than disadvantages. Doubts, worries, hesitation, and discrepancies were present alongside positive remarks. The degree of education has an impact on the availability of information, the pediatrician's care, and the sense of duty to get vaccinated
Migriño et al. [19]	2020	Philippines	Cross-sectional study	110 respondents	Not reported	SAGE group questionnaire	Vaccine hesitation determinants can vary greatly depending on context and environment, as seen by the absence of correlation between sociodemographic characteristics and vaccine hesitancy
Bocquier et al. [20]	2018	France	Cross-sectional survey	3,927 parents	January and July 2016	SAGE group’s questionnaire	Vaccine delay and refusal are common among French parents, particularly those with higher levels of education. Research indicates that trust and commitment levels are important factors in determining VH
Wang et al. [21]	2022	China	Cross-sectional study	5,102 parents	September 2020 to June 2021	WHO SAGE Vaccine Hesitancy tool	The COVID-19 vaccine is becoming more and more popular in Wuxi City, China. To allay public fears over the safety of vaccines, effective interventions are required
Masters et al. [22]	2018	Ethiopia	Cross-sectional study	350 caregivers	June 1-21, 2017	WHO SAGE Vaccine Hesitancy tool	Children's delayed immunization was highly correlated with high vaccine hesitation, suggesting that more efforts to inform clinicians and the community about vaccines may improve vaccine timeliness
Yakum et al. [23]	2022	Cameroon	Cross-sectional study	529 parents	November 2021	WHO SAGE Vaccine Hesitancy tool	Wealth has no bearing on vaccine reluctance, and in Yaounde, Cameroon, the main reason for vaccine hesitancy about routine vaccinations was a lack of trust
Ghosh et al. [24]	2022	India	Cross-sectional study	1,678 caregivers	June 2018 to November 2019	WHO SAGE Vaccine Hesitancy tool	To help public health care providers prioritize resources and concentrate on preventable measures such as health awareness, maintaining institutional births, and expanding free health-service delivery to increase immunization coverage, the study highlights the severity of the incomplete immunization, or VH, issue and identifies its contributing factors
Goruntla et al. [25]	2023	India	Cross-sectional study	574 respondents	July to December 2021	WHO SAGE Vaccine Hesitancy tool	The main reasons for parents' reluctance or refusal are kid safety and health. Policymakers have to decrease vaccine hesitancy by creating policies based on WHO-SAGE working group predictions and demographic data in order to reach 100% vaccination coverage
Khattak et al. [26]	2021	Pakistan	Cross-sectional study	610 parents	March to July 2019	WHO SAGE Vaccine Hesitancy tool	The high prevalence of vaccination rejection among parents was linked to food security, unemployment, mobile phone ownership, lack of education, and the inability to comprehend words or write
Hadjipanayis et al. [27]	2020	18 European countries	Mixed study	5,736 parents	Not reported	PACV and WHO SAGE Vaccine Hesitancy tool	The majority of parents in Europe believe in the importance of childhood vaccination. However, significant lack of confidence was found in certain European countries, highlighting the need for continuous monitoring, awareness, and response plans
Durmaz et al. [28]	2022	Turkey	Cross-sectional study	1,087 parents	September and December 2021	PACV scale	Public health policy can overcome obstacles and boost vaccination rates by comprehending the complex reasons behind vaccination hesitancy. Parents require information about vaccines, and the controversy related to COVID-19 vaccines can erode parents' trust in routine childhood immunizations
Yörük and Güler [29]	2021	Turkey	Cross-sectional study	370 parents	September to December 2020	PACV scale	In the upcoming years, it is important to keep a close eye on the prevalence of risk factors and vaccine reluctance
Thapar et al. [30]	2021	India	Cross-sectional study	172 mothers	March and April 2017	PACV scale	Mangalore's VH prevalence is extremely low when compared to comparable studies conducted in India and other countries. Due to worries about vaccine safety, a small percentage of participants had declined vaccination
Ustuner Top et al. [31]	2023	Turkey	Cross-sectional study	582 parents	July 2021 and October 2021	PACV scale	Regarding childhood vaccinations, 30% of the parents were hesitant. Digital literacy has a detrimental impact on vaccine hesitancy, but cyberchondria has a beneficial effect
Dubé et al. [32]	2019	Canada	Cross-sectional study	2,645 mothers of newborns	March 2014 to February 2015	PACV scale	Despite the fact that most moms had favorable opinions about vaccinations, many were either moderately or extremely hesitant about them
Williams et al. [33]	2021	United States	Cross-sectional study	263 parents	August 2019 to February 2020	PACV scale	We were unable to attract new dyads that did not show up for care because of a social desirability bias that may have resulted from trust among staff and patients
Sahoo et al. [34]	2023	India	Cross-sectional study	196 caregivers	March to May 2019	WHO SAGE and PACV scale	Even among caregivers who attend a tertiary care facility, concerns about vaccine reluctance are common. Therefore, more research is needed to evaluate hesitancy in remote, inaccessible, urban, and rural regions
Bianco et al. [35]	2019	Italy	Cross-sectional study	575 parents	April to June 2017	PACV scale	The findings of the study point to significant possible factors of VH, including attitudes toward prevention and the media and communication environments

The sample sizes of the included studies varied considerably, ranging from 110 participants in the study by Migriño et al. [19] to 5,102 participants in the study by Wang et al. [21]. This variation in sample size should be considered when interpreting the results of individual studies and the overall findings of the review. The study periods also varied, with some studies collecting data over a few months, e.g., February and July 2016 in Brown et al. [13], while others spanned over a year or more, e.g., November 2015 to April 2017 in Cherian et al. [16]. One study by Migriño et al. [19] did not report the study period. This difference in study duration could potentially influence the findings due to temporal changes in public perception and attitudes towards vaccination.

Several data collection tools were used across the included studies. Some studies employed validated questionnaires, such as the vaccine confidence index used by Brown et al. [13], the WHO Strategic Advisory Group of Experts on Immunization (SAGE) Vaccine Hesitancy tool [21-27,34], and the Parents Attitudes about Childhood Vaccines (PACV) scale [27-35]. Other studies utilized self-administered or self-structured questionnaires [14-18]. The use of standardized tools like the WHO SAGE and PACV scales allows for comparisons across different studies and populations. However, the use of self-structured questionnaires may introduce heterogeneity in the data collected, as these tools may not have undergone rigorous validation processes.

The key findings of the studies highlighted various aspects of vaccination hesitancy. Several studies reported a correlation between lower vaccine confidence and higher hesitancy [13,15]. Some studies identified specific factors associated with hesitancy, such as lower parental education [14], concerns about vaccine safety [15,30], lack of trust in healthcare providers [23], and lower socioeconomic status [26]. Other studies emphasized the role of effective communication strategies in addressing hesitancy [15,21] and highlighted the importance of healthcare professionals as trusted sources of information [17]. One study noted the influence of digital literacy and cyberchondria on vaccine hesitancy [31]. Some studies also reported on the prevalence of vaccine delay and refusal [20,22] and the impact of the COVID-19 pandemic on vaccine confidence [21,28]. The studies collectively underscore the complex and multifaceted nature of vaccination hesitancy and the need for tailored interventions to address this public health challenge.

Discussion

In this systematic review, 23 relevant studies concerning VH and its effects on immunization coverage within children were integrated. The cross-sectional research designs dominated the included studies and provided a wide geographical coverage, including European, Asian, African, and both American continents. The fact that the sample is diverse geographically is important, too, because cultural, socioeconomic, and healthcare practices affect perceptions of vaccines differently. One of the limitations we encountered included differences in the subject sample size from across the literature, the period of study in the various sources, and the variety of data collection tools applied. Nonetheless, such heterogeneity enhances the richness of the picture of VH as a phenomenon. This variability brings into the equations the notion that one is attempting to understand a phenomenon that is affected by multiple factors that may not work in a matrix called harmony.

One of the most common concepts that emerged from the discussed articles is the multifactorial approach to VH. The perception of safety was another category that came out clearly and was also affirmed in the literature review on vaccine hesitancy in other countries. These concerns are frequently based on information gathered through social media and community systems, highlighting the importance of efficient risk messaging and communication [36]. These issues must not only be answered but done so with proper, inclusive, and understandable information to establish credibility and promote empowered decision-making. As a fourth factor, confidence in the healthcare providers was identified as the other essential element in vaccine acceptance. Research showed that the level of parents' trust in healthcare professionals regarding information about vaccines was directly proportional to increased vaccination rates [37]. This shows that significant effort must be made toward building good patient-doctor communications with an easy ability to discuss parental concerns, along with the presence of empathy and thoroughness. Doctors and nurses are in a better place to offer vaccination recommendations and boost people’s confidence during the process [38].

Another crucial determinant of VH is inferred to be socioeconomic [39]. Several higher hesitancy rates and hesitancy associations in lower income countries were observed, including the educational level of parents, unemployment of parents, and dietary insecurity. Thus, these results evidence the paramount importance of social factors concerning vaccination perception and practice. When people experience poverty and lack money for food and shelter, illness is not a priority, nor is the ability to receive concrete information about how to prevent it. There is a need to address these fundamental social and economic determinants of health to enhance vaccine coverage and equity. Moreover, the cultures and traditions across various population groups constitute one of the most important contributions to vaccine acceptance [40]. Future interventions and programs in these regions must be created within a cultural context that is sensitive to social issues affecting each population. It can, therefore, be concluded that a one-size-fits-all type of intervention will not be efficient in intervening in the multiple factors that have been linked to VH.

The fact that many of the included studies employed different data collection instruments reduced the risk of heterogeneity but offered findings that were also informative. Due to the use of standardized tools, including the WHO SAGE Vaccine Hesitancy tool and the PACV scale, comparisons could be made across populations and other contexts of the research. These standardized tools are a more reliable way of measuring VH, and this makes it easy to notice patterns and trends all over the world. However, difficulties in some studies regarding the self-structuring of the questionnaires increase the requirements for other research, where the validated instruments should be used to improve the comparability of results of various researchers [20,28]. This would enable more significant meta-analyses and a clearer picture of global VH as a benefit of the proposed research.

The review also gave strong tendentious proof concerning the influence of VH on precise immunization coverage rates. Several studies described statistically significant relationships between increased levels of VH and impaired or incomplete vaccination schedules in childhood. This has serious consequences concerning both personal and population health and leads to a much higher probability of the emergence of the given VPDs [41]. Getting and sticking to high levels of immunization coverage is crucial for achieving herd immunity, which is essential for protecting other children who cannot be vaccinated on medical grounds, such as immunocompromised kids. Thus, VH elimination is not a luxury or an exercise in individual freedom, but a necessity on the part of population health.

The COVID-19 pandemic has made the issue of VH even more complicated. A few of the studies included in this review mentioned the decline in people’s trust in vaccines, which was associated with COVID-19 perceived risk regarding vaccines developed in record time. This underlines the importance of timely tracking of attitudes toward vaccines and explaining such risks in an attempt to restore people’s confidence in immunization. It also brought out the applicability of the peripheral components as a result of digital literacy in their perception of vaccines. The high uptake and rapid dissemination of sometimes false and misleading information on social media means that the prevention of the spread of such information requires specific programs that will be directed toward availing health literacy to the society and ensuring that the society comprehensively understands content constituents that are important in evaluating health information [42].

There are some limitations in our study. The frequent application of cross-sectional research also blunts the chances of proving cause-related relationships between VH and immunization coverage. Subsequent studies using more longitudinal approaches to study VH would be useful in descriptively charting the course of VH and its impact on future vaccination behaviors. Some of the weaknesses, which include the source of the study populations, the approach to data collection, and the study time frames used, also reduce the generalizability of results. In addition, the possibility of intervention effects by publication bias that elevated or statistically significant/positive findings are more likely to be published has to be considered.

However, despite these limitations, this systematic review provides much-needed information about the multifaceted and dynamic topic of VH and its effects on administrative efforts at increasing immunization coverage among children. The implications underlined in the present work are almost exclusively relevant to the necessity of using complex and comprehensive interventions involving the multiple factors that are deemed to cause VH. These interventions should prioritize several key areas: endorse and improve individual and collective communication between careers and parents; challenge misinformation constantly; educate on sustainable health; address structural factors that determine health; intervene to meet the needs of a particular community; and continue to supervise and evaluate this crucial field. By employing optimal analytic and preventive measures along with creating positive leadership and social accountability, VH will be effectively handled, and a larger initiative for the provision of high and equitable immunization among children will be achieved.

## Conclusions

Some of the causes of VH include perceived risks and complications of vaccines, doctors and other healthcare workers, economic reforms, cultural practices, and perceived rumors, myths, and misconceptions. These findings highlight the importance of addressing supply-side factors relating to clarity, recognizable evidence-based voice, campaign against falsehoods, and health literacy as well as demand factors on socioeconomic determinants. More observations and studies should be done regularly to establish measures of promoting high uptake of the vaccines by children.
